# Correlation between keratinized tissue width and periodontal indices around implant-supported fixed partial dentures

**DOI:** 10.15171/japid.2018.005

**Published:** 2018-06-20

**Authors:** Ilnaz Farhoudi, Soheil Parsay

**Affiliations:** ^1^Ardabil University of Medical Sciences, Ardabil, Iran; ^2^Private Practice, Ardabil, Iran

**Keywords:** Dental implants, keratinized tissue, peri-implant mucosa

## Abstract

**Background:**

The effect of keratinized tissue width on the peri-implant health has not been well elucidated. The results of previous studies on this topic are controversial and the role of keratinized tissue width in the long-term success of dental implants has not been confirmed. This cross-sectional study aimed to assess the correlation of keratinized tissue width with periodontal indices around implant-supported fixed partial dentures (FPDs).

**Methods:**

This cross-sectional study evaluated 73 implants. Patients underwent periodontal examinations, including measurement of plaque index (PI), gingival index (GI), clinical probing depth (PD), bleeding on probing (BOP), marginal gingival recession, keratinized mucosa width and radiographic marginal bone level. Data were analyzed using SPSS.

**Results:**

The mean GI, PI and marginal gingival recession around implants with <2 mm width of keratinized gingiva were greater than the corresponding values around implants with keratinized tissue width of ≥2 mm. However, this difference was not statistically significant (P>0.05). No significant differences were noted in PD and radiographic marginal bone level between the two implant groups with keratinized tissue width <2 mm and ≥2 mm. Thus, no correlation was found between the keratinized tissue width and the measured indices.

**Conclusion:**

Although this study did not show a significant correlation between the keratinized tissue width and peri-implant tissue health and consequently the implant success rate, long-term interventional studies are required to make a final judgment in this respect.

## Introduction

Dental implants are a reliable and predictable treatment option for replacement of the lost teeth, which can restore both esthetics and function. At present, dental implant treatment is highly popular due to its biological stability.^1^ The significance of keratinized mucosa around dental implants has been a topic of debate in the literature.^2^ Due to structural and anatomical differences between teeth and implants, presence of healthy soft tissue around dental implants seems to be more important than around natural teeth. Moreover, disintegration and recession of soft tissue around dental implants occurs faster and is more severe compared to that around natural teeth.^3^ Junctional epithelium and healthy connective tissue around teeth are the first line of defense against microbial invasion, and adequate biologic seal is the cornerstone of dental implant success.^4^

Supracrestal collagen fibers are vertically oriented relative to the tooth surface and are attached to the cementum covering the root surface. However, these fibers are oriented parallel around dental implants.^3,5,6^ The biologic width around dental implants is 3‒4 mm, which is composed of junctional epithelium and the connective tissue fibers, which are positioned parallel to the implant surface.^4^ In an observational study, Loe and Lang suggested 2 mm of keratinized tissue width, including 1 mm of attached gingiva around dental implants.^7^ In a cross-sectional study aiming to determine the ideal width of keratinized mucosa around natural teeth and the protective capacity of the mucosa, the authors concluded that inflammation remains if the oral hygiene remains poor, irrespective of the mucosal width of >2 mm or ≤2 mm. However, follow-up examinations at 5 years revealed higher accumulation of plaque and inflammation in the absence of keratinized gingiva. Dental implants with attached gingiva <2 mm are more prone to gingival recession and bone loss. In prosthetic treatments with limitations with regard to extension into the gingival sulcus, a minimum of 5 mm of keratinized gingiva width is necessary because such restorations enhance plaque accumulation and gingival inflammation in areas with keratinized tissue width <2 mm.^4^ Evidence shows less plaque accumulation, tissue inflammation and gingival recession around dental implants in the presence of adequate amount of keratinized tissue. However, some others have shown that the peri-implant tissue health can be maintained with adequate oral hygiene even in the absence of keratinized tissue and there is no significant correlation between keratinized tissue width and peri-implant soft tissue health.^8^

Soft tissue condition and implant health may be variable in different implant-supported fixed partial dentures (FPDs) and might affect their maintenance, durability and success rate. Considering the controversy in the results of previous studies and to determine the factors related to peri-implant gingival health, this study aimed to assess the correlation of keratinized tissue width and periodontal indices around implant-supported FPDs.

## Methods

The target population of this cross-sectional study comprised of patients with implant-supported FPDs one year after their prosthetic delivery. A total of 73 implants were evaluated.

All the patients were thoroughly informed of the aims of the study and processes of examination, and written informed consent was obtained from them. Data regarding age, gender and periodontal indices were collected. A parallel periapical radiograph was obtained from implant sites to assess alterations in bone around dental implants. These examinations included plaque index (PI), gingival index (GI), bleeding on probing (BOP), clinical probing depth (PD), marginal gingival recession, width of keratinized mucosa and radiographic marginal bone level.

The PI was assessed using the Silness and Loe plaque index. The amount of plaque covering the surface of crowns in four areas of mesiobuccal, mid-buccal, distobuccal and lingual/palatal was assessed and scored from 0 to 3. The scores of the four areas were added and divided by 4 to obtain the mean score for each implant. According to the Silness and Loe PI, 0 indicated absence of plaque, 1 indicated a low amount of plaque, 2 indicated a moderate amount of plaque and 3 indicated a high amount of plaque.^9^

The GI was determined using the Loe and Silness GI. Gingival tissue was assessed at four points around dental implants (mesiobuccal, mid-buccal, distobuccal and lingual/palatal) in terms of the presence of inflammation and scored from 0 to 3. The scores were summed and divided by four to obtain the mean value for each implant. According to the Loe and Silness GI, 0 indicated natural gingiva, 1 indicated mild inflammation, 3 indicated moderate inflammation and 4 indicated severe inflammation.^9^

For assessment of BOP, the periodontal probe was inserted into the gingival sulcus and was walked around the implant with a certain pressure. Bleeding was assessed after 30 seconds: 0 indicated no bleeding (negative) and 1 indicated bleeding (positive).^10^

For assessment of PD, the distance from the gingival margin to the sulcus depth was measured at four pints of mesiobuccal, mid-buccal, distobuccal and lingual/palatal around each implant using a Williams probe and reported in millimeters. The mean of the four values was considered as the mean PD.^9^ For assessment of marginal gingival recession, the finishing line of the crown served as the cementoenamel junction of natural teeth and as in natural teeth, the distance from this line to gingival margin was considered as the amount of gingival recession and reported in millimeters.^10^

Radiographic marginal bone level was defined as the vertical distance from the implant border to the first implant-bone contact point at the mesial and distal aspects on parallel digital periapical radiographs taken with a photostimulable phosphor plate detector.

Considering the ratio of implant height to its radiographic image, radiographic magnification was determined and accordingly, actual values were calculated.

In cases where primary radiographs were not available, implant border was considered bone-level at the time of surgery and bone remodeling within the first year was considered to be 1 mm according to a similar study.^11^

Keratinized mucosa width was defined as the distance between the gingival margin and mucogingival junction at the mid-buccal area, which was measured by a Williams probe with 1 mm accuracy.^10^

The inclusion criterion was patients with implant-supported FPDs, in which at least one year had passed since their prosthetic delivery and loading. The exclusion criteria consisted of cigarette smoking, pregnancy, antibiotic use in the past six months, systemic conditions requiring antibiotic prophylaxis, and systemic diseases affecting bone metabolism and soft tissue such as hyperthyroidism, hyperparathyroidism and uncontrolled diabetes mellitus.

All data were collected and analyzed by t-test and chi-squared test using SPSS 20.

## Results

The periodontal indices were compared between the two groups with keratinized mucosa width <2 mm and ≥2 mm around dental implants. The results showed no significant difference in marginal gingival recession between the two groups (P>0.05).

No significant difference was noted in radiographic marginal bone level, PD in different areas or the mean PD between the two groups (P>0.05). The mean amount of GI was 1.36 ± 0.84. The correlation between KM and GI was not statistically significant (P=0.09) and the mean amount of PI was 1.17 ± 0.8 and also the correlation between PI and KM was not statistically significant. (P=0.78) The correlation between BOP and keratinized mucosa width was not statistically significant too (P=0.9).

The comparison of PD, radiographic marginal bone level and marginal gingival recession in the two groups are shown in Table 1.

**Table 1:** Comparison of PD, radiographic marginal bone level and marginal gingival recession in the two groups with keratinized mucosa width <2 mm and ≥2 mm

**Index**	**Keratinized mucosa width**	**Number**	**Mean**	**Standard deviation**	**P-value**
**Mean radiographic marginal bone level**	≥2 mm	53	0.79	0.61	0.79
	<2 mm	20	0.76	0.42	
**Mean probing depth of the four areas**	≥2 mm	53	3.50	1.20	0.08
	<2 mm	20	2.90	1.48	
**Marginal gingival recession**	≥2 mm	53	0.68	0.75	0.072
	<2 mm	20	1.05	0.83	

## Discussion

A consensus has not been reached by the experts regarding the significance of the presence of keratinized gingiva around dental implants. There is no evidence to support the need for the presence of keratinized gingiva around dental implants.^12^ Lang and Loe claimed that 2 mm of keratinized gingiva and 1 mm of attached gingiva are required for gingival health. Prospective studies have shown that if the patient adheres to oral hygiene instructions, long-term health of the hard and soft tissue will not be compromised even in the absence of keratinized tissue.^13^

Theoretically, peri-implant soft tissue is more sensitive to inflammation and bone loss than the soft tissue around natural teeth due to structural differences such as less blood supply, fewer fibroblasts and no attachment of tissue to cementum.

This study aimed to assess the correlation of keratinized tissue width and periodontal parameters (determined by clinical and radiographic examinations) around implant-supported FPDs. The study hypothesis was that a significant association exists between keratinized tissue width around dental implants and gingival health parameters and consequently the success of implant-supported FPD.

First, periodontal health indices such as PI, GI, BOP, PD, keratinized mucosa width, and marginal gingival recession were clinically measured. The patients were then requested to take parallel digital periapical radiographs using a PSP detector. Radiographic marginal bone level was assessed on parallel periapical radiographs.

Chang et al^14^ evaluated 239 implants in 69 patients that had been loaded for 3‒4 years. They measured BOP, PD, GI, PI and keratinized mucosa width and evaluated pre- and post-operative radiographs to assess bone resorption. In their study, PI and GI were significantly higher in patients with keratinized mucosa width of <2 mm. In our study, different results were found regarding GI and PI. However, the main difference between their study and ours was the variability in implant brands used in their study. They also evaluated straight implants, which would definitely affect the PI and GI.

Considering the results of this study, clinical PI was significantly better in implant-supported FPDs with keratinized mucosa width of ≥2 mm, which might be attributed to patients’ superior oral hygiene and better oral hygiene control in areas with keratinized mucosa width of ≥2 mm. However, no significant difference was noted in GI, marginal gingival recession, BOP, radiographic marginal bone level and PD between the two groups with keratinized mucosa width of ≥2 mm and <2 mm.

Ladwein et al^2^ evaluated the association of the presence of keratinized mucosa around dental implants and gingival health and found no significant differences in PD and radiographic vertical bone levels between the two groups with and without keratinized mucosa. But PI and BOP were greater around implants without keratinized mucosa. Thus, keratinized mucosa seems to have a significant effect on peri-implant gingival health but does not seem to affect the level of peri-implant bone.^2^

Esfahanian et al^15^ assessed the correlation of keratinized tissue width and periodontal parameters around implant-supported FPDs and showed that increased width of keratinized gingiva and attached gingiva around implants is not necessarily associated with higher level of peri-implant health. Bouri et al^5^ assessed the association of keratinized mucosa width and health status of the peri-implant soft tissue and reported that increased width of keratinized gingiva around dental implants is associated with lower mean bone resorption and improved soft tissue indices.

Esper et al^4^ evaluated fixed dental implants placed at the site of cleft in patients with cleft lip and palate in terms of clinical parameters such as PD, PI and GI. The results showed that all the clinical parameters had a significant correlation with keratinized tissue width around dental implants.

Adibrad et al^16^ evaluated functional dental implants in terms of periodontal parameters, including GI, PI, BOP, PD, marginal gingival recession, periodontal attachment loss, radiographic marginal bone level and keratinized tissue width and reported that keratinized mucosa width had no significant association with bone loss around dental implants. Absence of adequate keratinized tissue width around dental implants is associated with higher levels of PI, GI, BOP and marginal gingival recession.

Epozita et al^17^ in a meta-analysis showed that soft tissue health in terms of GI affects the health of posterior implants. They concluded that implant position plays a more effective role than the keratinized mucosa because they reported that annual bone resorption in posterior implants is 3.5 times the rate in anterior implants. Assessment of GI and marginal bone loss in the current study was not performed in terms of the implant position. This was a limitation of this study and it is suggested that it should be performed in future studies.

## Conclusion

According to the results of the current study and those of previous studies, presence of adequate keratinized tissue around dental implants can improve gingival health indices. However, absence of adequate keratinized mucosa does not necessarily mean that the health of the surrounding tissue is compromised or the implant success is at risk. Some other factors such as oral hygiene also profoundly affect the gingival health. An ideal oral hygiene in an area with a narrow or no keratinized mucosa might be associated with normal bone and gingival indices. In an area with wide keratinized mucosa and poor oral hygiene, gingiva and bone health might be compromised.

## Authors’ contributions

The study was planned by IF and SP. Data collection statistical analyses and interpretation of data was carried out by IF and SP. The manuscript was prepared by IF and SP. All the authors have read and approved the final manuscript for submission.

## Competing interests

The authors declare that they have no competing interests.

## Ethics approval

This study was approved by the Ethics Committee of Ardabil University of Medical Sciences under the code IR-ARUMS REC-1396-35.
